# Prepubertal start of father's smoking and increased body fat in his sons: further characterisation of paternal transgenerational responses

**DOI:** 10.1038/ejhg.2014.31

**Published:** 2014-04-02

**Authors:** Kate Northstone, Jean Golding, George Davey Smith, Laura L Miller, Marcus Pembrey

**Affiliations:** 1 School of Social and Community Medicine, University of Bristol, London, UK; 2 Centre for Child and Adolescent Health, University of Bristol, London, UK; 3 MRC Integrative Epidemiology Unit at the University of Bristol, UK; 4 UCL Institute of Child Health, London, UK

## Abstract

Despite interest in the idea that transgenerational effects of adverse exposures might contribute to population health trends, there are few human data. This non-genetic inheritance is all the more remarkable when transmission is down the male-line as reported in a historical Swedish study, where the paternal grandfather's food supply in mid childhood was associated with the mortality rate in his grandsons. Using the Avon Longitudinal Study of Parents and Children's questionnaire data on smoking and smoking onset from 9886 fathers, we examined the growth of their children from 7–17 years. Adjusting for potential confounders, we assessed associations between body mass index (BMI), waist circumference, total fat mass and lean mass with the age at which the father had started smoking regularly. Of 5376 fathers who reported having ever smoked, 166 reported regular smoking <11 years of age. Before adjustment, those offspring whose fathers started smoking <11 years had the highest mean BMIs at each age tested. The adjusted mean differences in BMI, waist circumference and total fat mass in those sons whose fathers started smoking <11 years, compared with all other sons, increased with age, being significantly greater from 13 years onwards. There were no significant BMI associations in daughters, but they showed a reduction in total lean mass. Our results highlight the importance of the developmental timing of the paternal exposure as well as gender differences in offspring outcomes. Smoking by boys in mid childhood may contribute to obesity in adolescent boys of the next generation.

## Introduction

There is a long history of experimental demonstration of transgenerational effects from an ancestral exposure such as toxins, drugs or surgically induced diabetes in mammals, including sex-specific transmissions of phenotypic effects over several generations.^1, 2, 3, 4, 5^ Some studies have focused on imprinted gene expression in descendants^6, 7^ and others on associated epigenetic changes,^8, 9^ but no transgenerational signal itself has been clearly defined.^10^

In consideration of the current rise in the prevalence of obesity, it is important to bear in mind that some determinants may have been operating in the previous generation(s) with dietary and lifestyle exposures initiating changes, possibly adaptive epigenetic changes, in germline cells.^6, 7, 11, 12^ However, in human transgenerational studies, it can be difficult to separate the effect of parental or ancestral environmental exposures from: (a) social patterning across the generations, (b) parental genetic makeup or (c) direct maternal effects via the oocyte or placenta. However, male-line transmissions that are only induced during particular exposure-sensitive periods in development go some way to dealing with social confounding. For example, historical studies in Sweden have shown an association between the paternal grandfathers' food supply in mid childhood (few years before the prepubertal growth spurt) and longevity and deaths from diabetes in their grandchildren.^13, 14^ Subsequent analysis by sex of the grandchildren showed that the paternal grandfathers' food supply was linked only to the mortality rate in grandsons and only for grandpaternal exposure between 7 and 11 years and not in adolescence.^15^

In consequence, we initiated the current study on the transgenerational effect of the onset of paternal smoking, hypothesising that if there were a transgenerational effect it would be confined to smoking onset during mid childhood before puberty and not later. As the age of puberty has decreased over time, we considered the time period to be <11 years. Preliminary results following the offspring to 9 years were in the direction of the hypothesis^15^ and we now report an extended analysis with follow-up of the offspring to 17 years.

## Subjects and methods

The Avon Longitudinal Study of Parents and Children (ALSPAC, see website: www.bristol.ac.uk/alspac) recruited 14 541 pregnant women resident in Avon, UK with expected dates of delivery between 1st April 1991 and 31st December 1992.^16^ The pregnant woman could invite her partner to take part if she chose to. For almost 10 000 pregnancies, a questionnaire was completed by the father during pregnancy. The fathers were asked: ‘Have you ever been a smoker?' 5451 of the 9886 fathers who answered this, responded positively; a subsequent question asked if so, ‘at what age did you start smoking regularly?' A later question asked was whether the father was smoking at around the time of conception.

Children were measured using standardised methods by the ALSPAC study team in a clinic setting from the age of 7 and every other year thereafter until the age of 17 (*n*=6116 with paternal smoking information at age 7 and *n*=3740 at 17); body mass index (BMI) was calculated as weight (kg)/height(m)^2^. Waist circumference was measured at each time point except 17 years of age. Total-body fat mass was measured from the age of 9 using total-body dual-energy X-ray absorptiometry (DXA) scans, performed using a Lunar Prodigy dual-energy X-ray absorptiometer (GE Medical Systems Lunar, Madison, WI, USA).^17^ For girls, age at menarche was assessed via annual questionnaires from age 8 onwards. For boys, annual questions were asked about various indicators of puberty, including stage of growth of pubic hair. Here, we used the assessment at age 11 years.

Differences in mean BMI scores according to categories of the age at which the father started smoking (categorised as <11 years, 11–12 years, 13–14 years, 15+ years) were examined using ANOVA. In addition, we compared mean waist circumference and body fat mass in those whose fathers started smoking <11 years of age with the rest of the population (including those who never smoked). Analyses were then adjusted for parity of the mother at the time of birth of the offspring (primiparae *vs* multiparae), highest maternal education level (using five categories from ‘low' to ‘university degree'), housing tenure (owner–occupier; public rented; other rented), maternal smoking during pregnancy (yes *vs* no) and paternal smoking at conception (yes *vs* no). Similar methods were used for analyses of waist circumference, fat mass and lean mass. We repeated the regression analysis for BMI but using maternal age at start of regular smoking. All analyses were performed using SPSS (Version 21.0, Armonk, NY, USA; IBM Corp).

## Results

### Paternal onset of regular smoking

In the ALSPAC study, the 5376 fathers who had ever smoked reported the age at which they had started smoking regularly: the most common age of onset was 16 years, but 166 (3% of the ever smokers) reported regular smoking before age 11 when most would be prepuberty. Table 1 shows that there is no difference in the ages at which the father started smoking in regard to the gender of the offspring.

In general, the mean BMI increases with the age of the study child at measurement, the daughters having consistently higher mean BMI compared with the sons (Supplementary Table 1). Table 2 demonstrates that, before adjustment, those offspring whose fathers started regular smoking before the age of 11 had the highest mean BMIs at each age tested, and that the difference between the growth of those whose fathers started smoking at <11 years and those who started at later ages increased as the study child got older.

**Figure 1:**
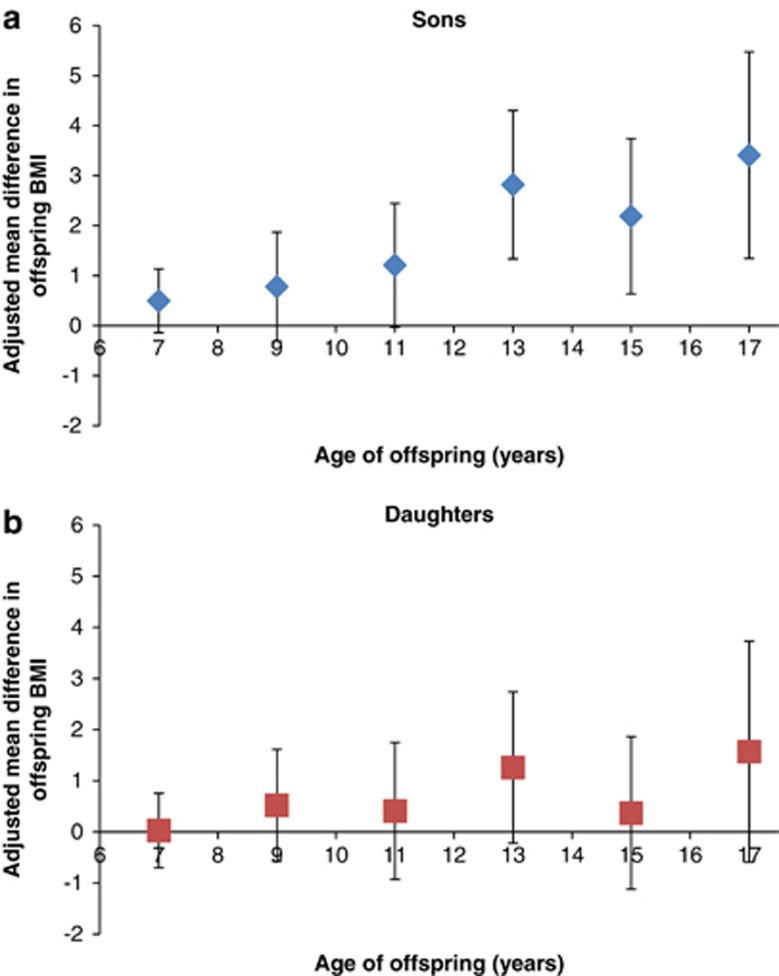
(**a** and **b**) Adjusted (adjusted for parity, parental education, maternal smoking during pregnancy, housing tenure and paternal smoking at conception) mean difference in offspring BMI (95% confidence intervals) for those whose fathers started smoking regularly <11 years of age compared with those who either did not smoke or did not start until 11 years of age or older for (**a**) sons and (**b**) daughters.

To test our overarching hypothesis that offspring of fathers who started smoking before age 11 would be more overweight, we compared, for each gender, the differences in mean BMIs adjusted for the potential confounders outlined in the methodology (including whether or not the father was smoking at conception as that had been shown to be associated with childhood BMI^18^), comparing those offspring whose fathers started smoking before the age of 11 with the rest of the population (Supplementary Table 2). The adjusted mean differences in BMI, waist circumference and fat mass in the group of children whose fathers started smoking <11 years compared with the rest tend to increase as the children got older, and showed significant increases in all measures at ages 13, 15 and 17. The relationships were then calculated for each sex. It can be seen (Figures 1a and b) that, compared with all other study children, the sons whose fathers had started smoking early (<11) had greater mean BMI when measured after the onset of puberty (≥11). Although the girls also had greater mean differences at some age points in adolescence, these were consistently less than those of the boys and did not reach statistical significance.

Further analysis of other markers of body size (waist circumference and fat and lean body mass) of each group are shown in Table 3. It can be seen that the mean waist circumferences of the adolescent boys (aged 13–15) whose fathers had started regular smoking <11 exceeded those of the rest of the population by about 4.8 cm. For mean fat mass, the findings were more dramatic: the sons were found to have markedly increased levels of fat mass computed from whole-body DXA scans, ranging from an excess of between 5 and 10 kg body fat between ages 13 and 17. Although there was a suggestion of excess waist circumference and fat mass in the daughters, the effects were less consistent. Lean mass showed no excess among the sons – but for the daughters there were significant reductions between ages 9 and 13.

### Maternal age at onset of regular smoking

We determined whether a similar effect on body size was present for the age at which the study mothers had started smoking regularly; very few mothers reported smoking before the age of 11 (1% of smokers), and their offspring showed no evidence of an increase in mean BMI. There were no differences for either their sons or their daughters at any of the ages at which their mother had started smoking (Supplementary Table 3).

### Assessment of possible explanations for our findings

Further analyses tested whether the children whose fathers started smoking <11 had an earlier onset of puberty (as children with early puberty tend to become more overweight in adolescence), but we found no differences in relation to age at onset of smoking (Supplementary Tables 4a, b). We tested whether the fathers who started smoking early had higher mean BMIs themselves, but on the contrary, these men tended to have lower mean BMIs, compared with all other groups (Supplementary Table 5). Forty-six per cent of the sons of fathers who started smoking <11 had themselves started smoking by 17; however, there was no correlation of the sons' smoking status or age of onset of smoking with their BMI (Supplementary Table 6), and taking their own smoking status into account (Supplementary Table 7) confirmed the findings shown in Table 3. Further analyses to determine the specificity of our findings showed that the male offspring of the fathers who started to smoke between ages 11 and 13 did not have such striking increases in fat mass or waist circumference as those whose fathers started smoking <11 (Supplementary Table 8).

In order to determine whether there were indicators of genetic differences in the children of fathers who started smoking early that would predispose them to increased BMI, we examined genotypes available in ALSPAC that are associated with phenotypic variability in BMI. We considered variant rs9939609 in the FTO gene, which has been shown to be related to BMI,^19, 20^ SNP rs1051730 at CHRNA5-CHRNA3-CHRNB4 that has been shown to interact with smoking to influence (decrease) BMI,^21^ as well as an adiposity allele score comprising 32 SNPs associated with BMI.^22^ None of these showed any significant relationship with the age at onset of the father's smoking (Supplementary Tables 9 and 10).

## Discussion

In support of our hypothesis, we have found an association between the onset of regular paternal smoking before the age of 11 years and raised BMI in adolescence (corroborated by increased mean waist circumference and whole-body fat mass) in their sons. This is the first thorough demonstration of a transgenerational effect of paternal smoking in mid childhood on his future offspring's body fat. Importantly, in line with the exposure-sensitive period found in other studies,^13, 15^ the transgenerational effect was observed among fathers who had started smoking before 11 years. This finding makes genetic pleiotropy, namely a transmitted DNA variant that both made the father more likely to smoke early and the son to over eat, an unlikely explanation for two reasons: (a) if early-smoking fathers had a gene variant that predisposed to both early smoking and adolescent obesity, one would expect early-smoking fathers themselves to have greater BMIs as adults (but actually they have lower BMI than expected); conversely, if 50% of the sons inherited this gene variant from their father, we would expect at least 50% to start smoking early, and for these sons to have greater BMI – a correlation we do not see; (b) the action of the theoretical pleiotropic allele would have to be remarkably developmentally stage dependent for it to explain the difference in BMI between the sons of fathers who started smoking <11 years compared with those who started smoking later. This is not to say that the response to the transgenerational signal in the offspring might not be influenced by their genetic makeup, but we found no significant difference in the distribution of a variety of genetic markers that have been shown to relate to increases in BMI.

There are a number of weaknesses in this study. First, the numbers of individuals whose fathers had started regular smoking prepuberty was small, and thus the confidence intervals are wide; nevertheless, significant associations were obtained with particularly striking effect sizes for body fat mass. Second, the response rates in this group, particularly for the boys, were low in adolescence (eg, the numbers attending for examination fell from 38 at age 7 to 14 at age 17); however, if this were to bias the results, most of the non-responders would need to have a relatively low BMI. In fact, at age 7, those who failed to respond later had a larger mean BMI; 16.40 *versus* 16.14 in those who went on to attend at 17 years (*P*≤0.0001).

The strengths of the study include the fact that it is set within a comprehensive contemporary birth cohort, which has allowed social and other potential confounders to be taken into account, including paternal smoking at conception of the study child. Second, the present study was initiated as a replication of the key findings of a Swedish study of the transgenerational impact of ancestral food supply:^13, 14, 15^ namely male-line transmission and an exposure-sensitive period before, but not during, puberty. The latter feature is difficult to infer meaningfully from rodent experiments. We have shown that there is a pronounced effect on children of the timing of this exposure in their fathers, and that there is no such effect of early initiation of smoking in the mothers. We have also shown that the effect on BMI and body fat is most pronounced in the sons, but cannot rule out a smaller effect in the daughters.

Cigarette smoking is a well-studied exposure known to be associated with long-lasting biological effects,^23^ but to our knowledge there are no human studies of the effect of paternal smoking in childhood on his offspring's metabolic development. However, a human study, testing what had been demonstrated earlier in mice,^2^ did find a dosage-dependent association of paternal betel quid use with early metabolic syndrome in the adult offspring who had never chewed betel quid themselves.^24^ Recent human studies have assessed paternal smoking and lifestyle on markers of DNA damage/instability in the cord blood of their offspring. Using tests for single and doubled strand DNA breaks in parental blood cells, spermatozoa and cord blood from 39 family trios, Laubenthal *et al*^25^ identified transgenerational DNA alterations in the unexposed offspring of smoking-exposed fathers. Paternal lifestyle, particularly smoking, has also been shown to be associated with increased germline DNA mutation rate at the unstable minisatellite locus CEB1 using 78 parent–child trios.^26^ The authors discuss this result in terms of mutation, but there is evidence from transgenerational studies of X-irradiation in mice that the increased minisatellite mutation rate in descendants is probably a marker of a cellular response to genotoxic stress, rather than a direct effect, as it was observed on chromosomes that were never irradiated.^27^ Relevant to our findings and potential mediating mechanisms is the report of hypomethylation at the imprinted gene IGF2 (differentially methylated region) in umbilical cord blood being associated with paternal obesity. This suggests a preconceptional impact of the obesity (and/or exposures related to it) on the reprogramming of imprint marks during spermatogenesis.^6^

Here, we have shown that cigarette smoking in mid childhood represents a valuable model for future analysis of human transgenerational responses both in terms of molecular mechanisms and public health implications.

## Figures and Tables

**Table 1:** Age at which the father started smoking regularly by gender of offspring

*Age at which the father started smoking*	*Sons*	*Daughters*
<11	89 (1.7%)	77 (1.6%)
11–12	185 (3.6%)	187 (3.8%)
13–14	508 (9.9%)	446 (9.2%)
15+	2013 (39.0%)	1871 (38.4%)
Never	2362 (45.8%)	2291 (47.0%)
Total	5157 (100%)	4516 (100%)

**Table 2:** Unadjusted mean (SD) offspring body mass index (kg/m^2^) at various ages by age father started smoking

	*Age 7*		*Age 9*		*Age 11*		*Age 13*		*Age 15*		*Age 17*	
*Age father started smoking*	n	*Mean (SD)*	n	*Mean (SD)*	n	*Mean (SD)*	n	*Mean (SD)*	n	*Mean (SD)*	n	*Mean (SD)*
*Sons*												
<11	38	16.5 (2.24)	28	17.9 (3.55)	30	19.8 (3.87)	22	22.3 (4.72)	20	23.1 (3.99)	14	25.9 (5.52)
11–12	82	16.3 (2.60)	72	17.9 (3.48)	73	19.3 (3.96)	56	20.7 (3.91)	44	21.1 (2.91)	36	22.5 (2.73)
13–14	241	16.0 (1.80)	228	17.8 (3.15)	203	19.3 (3.74)	167	20.1 (3.48)	140	21.4 (3.59)	125	23.3 (4.02)
15+	1188	16.0 (1.85)	1082	17.4 (2.61)	1019	18.6 (3.18)	872	19.8 (3.76)	730	20.9 (3.31)	625	22.4 (3.76)
Never	1555	15.9 (1.79)	1428	17.3 (2.56)	1337	18.6 (3.12)	1195	19.6 (3.17)	1041	20.8 (3.23)	853	22.4 (3.61)
												
*Daughters*												
<11	35	16.8 (2.21)	31	18.9 (3.28)	28	20.1 (3.31)	24	22.3 (3.56)	25	22.9 (3.37)	16	26.5 (5.48)
11–12	104	16.3 (2.37)	99	18.2 (3.48)	92	19.5 (3.89)	177	21.1 (4.08)	81	22.4 (4.54)	66	23.2 (5.06)
13–14	246	16.6 (2.35)	220	18.4 (3.09)	201	19.9 (3.58)	71	21.4 (3.68)	162	22.6 (3.76)	155	23.7 (4.47)
15+	1113	16.4 (2.24)	1064	17.9 (3.12)	1003	19.3 (3.62)	868	20.6 (3.48)	787	21.8 (3.83)	762	22.9 (4.20)
Never	1514	16.2 (1.95)	1450	17.6 (2.69)	1362	19.1 (3.24)	1213	20.5 (3.26)	1118	21.5 (3.26)	1088	22.7 (3.87)

**Table 3:** Adjusted^a^ mean difference (Md) (95% CI) in (a) waist circumference and (b) fat and lean mass assessed by DXA of the offspring if their father started smoking regularly <11 years of age (those who either did not smoke or did not start until 11 years of age or older are the reference group)

	*Age 7*	*Age 9*	*Age 11*	*Age 13*	*Age 15*	*Age 17*
*Sons*						
Waist circumference						
N^b^	33	24	27	19	18	Not collected
Md (95% CI)	+0.57 (−0.98, 2.12)	1.14 (−1.65, 3.94)	2.55 (−0.88, 5.98)	4.83 (0.98, 8.68)	4.84 (0.99, 8.66)	
P	0.470	0.423	0.123	0.014	0.006	
Fat mass						
N^b^	Not collected	23	26	19	17	13
Md (95% CI)		0.88 (−0.93, 2.69)	1.91 (0.65, 4.47)	5.79 (2.67, 8.91)	5.50 (1.88, 9.30)	10.6 (5.40, 15.9)
P		0.340	0.144	<0.0001	0.004	<0.0001
Lean mass						
N^b^	Not collected	23	26	19	17	12
Md (95% CI)		0.20 (−1.04, 1.43)	−0.61 (−2.28, 1.07)	0.42 (−2.84, 3.68)	−0.07 (−3.30, 3.16)	0.82 (−2.80, 4.44)
P		0.753	0.088	0.801	0.549	0.657
						
*Daughters*						
Waist circumference						
N^b^	35	31	28	24	25	Not collected
Md (95% CI)	1.29 (−0.45, 3.02)	3.36 (0.61, 6.11)	0.96 (−2.41, 4.34)	5.12 (1.66, 8.66)	2.86 (−0.74, 6.46)	
P	0.147	0.017	0.578	0.004	0.119	
Fat mass						
N^b^	Not collected	26	26	21	19	16
Md (95% CI)		1.84 (0.03, 3.66)	1.09 (−1.38, 3.55)	2.70 (−0.32, 5.73)	0.77 (−2.68, 4.22)	5.75 (1.25, 10.2)
P		0.046	0.388	0.080	0.662	0.012
Lean mass						
N^b^	Not collected	26	26	21	19	15
Md (95% CI)		−1.44 (−2.63, −0.21)	−2.04 (−3.80, −0.29)	−1.97 (−3.73, −0.21)	−1.62 (−3.45, 0.22)	−1.75 (−3.96, 0.47)
P		0.022	0.023	0.028	0.084	0.122

Abbreviations: CI, confidence interval; DXA, dual-energy X-ray absorptiometry.

a Adjusted for parity, parental education, maternal smoking during pregnancy, housing tenure and paternal smoking at conception.

b Numbers whose fathers started smoking regularly before age 11.

## References

[bib1] Campbell JH Perkins P Transgenerational effects of drug and hormone treatments in mammals: a review of observations and ideas Prog Brain Res 1988 73 535 553 3047810 10.1016/S0079-6123(08)60525-7

[bib2] Boucher BJ Ewen SW Stowers JM Betel nut (*Areca catechu*) consumption and the induction of glucose intolerance in adult CD1 mice and in their F1 and F2 offspring Diabetologia 1994 37 49 55 8150230 10.1007/BF00428777

[bib3] Anway MD Cupp AS Uzumcu M Skinner MK Epigenetic transgenerational actions of endocrine disruptors and male fertility Science 2005 308 1466 1469 15933200 10.1126/science.1108190 PMC11423801

[bib4] Franklin TB Russig H Weiss IC Epigenetic transmission of the impact of early stress across generations Biol Psychiatry 2010 68 408 415 20673872 10.1016/j.biopsych.2010.05.036

[bib5] Ng SF Lin RC Laybutt DR Barres R Owens JA Morris MJ Chronic high-fat diet in fathers programs β-cell dysfunction in female rat offspring Nature 2010 467 963 966 20962845 10.1038/nature09491

[bib6] Soubry A Schildkraut JM Murtha A Paternal obesity is associated with IGF2 hypomethylation in newborns: results from a Newborn Epigenetics Study (NEST) cohort BMC Med 2013 11 29 23388414 10.1186/1741-7015-11-29 PMC3584733

[bib7] Dunn GA Bale TL Maternal high-fat diet effects on third-generation female body size via the paternal lineage Endocrinol 2011 152 2228 2236 10.1210/en.2010-1461 PMC3100614 21447631

[bib8] Carone BR Fauquier L Habib N Paternally induced transgenerational environmental reprogramming of metabolic gene expression in mammals Cell 2010 143 1084 1096 21183072 10.1016/j.cell.2010.12.008 PMC3039484

[bib9] Burdge GC Slater-Jefferies J Torrens C Phillips ES Hanson MA Lillycrop KA Dietary protein restriction of pregnant rats in the F0 generation induces altered methylation of hepatic gene promoters in the adult male offspring in the F1 and F2 generations Br J Nutr 2007 97 435 439 17313703 10.1017/S0007114507352392 PMC2211514

[bib10] Daxinger L Whitelaw E Understanding transgenerational epigenetic inheritance via the gametes in mammals Nat Rev Genet 2012 31 153 162 22290458 10.1038/nrg3188

[bib11] Waterland RA Travisano M Tahiliani KG Rached MT Mirza S Methyl donor supplementation prevents transgenerational amplification of obesity Int J Obesity (Lond) 2008 32 1373 1379 10.1038/ijo.2008.100 PMC2574783 18626486

[bib12] Zaina S Lund G Paternal transmission, cardiovascular risk factors and epigenetics Curr Opin Lipidol 2012 23 586 587 23160405 10.1097/MOL.0b013e32835918cd

[bib13] Bygren LO Kaati G Edvinsson S Longevity determined by ancestors' over nutrition during their slow growth period Acta Biotheor 2001 49 53 59 11368478 10.1023/a:1010241825519

[bib14] Kaati G Bygren LO Edvinsson S Cardiovascular and diabetes mortality determined by nutrition during parents' and grandparents' slow growth period Eur J Hum Genet 2002 10 682 688 12404098 10.1038/sj.ejhg.5200859

[bib15] Pembrey ME Bygren LO Kaati G Sex-specific, male-line transgenerational responses in humans Eur J Hum Genet 2006 14 159 166 16391557 10.1038/sj.ejhg.5201538

[bib16] Boyd A Golding J Macleod J Cohort profile: the ‘Children of the 90s'—the index offspring of the Avon Longitudinal Study of Parents and Children Int J Epidemiol 2013 42 111 127 22507743 10.1093/ije/dys064 PMC3600618

[bib17] Toschke AM Martin RM von Kries R Wells J Davey Smith G Ness AR Infant feeding method and obesity: body mass index and dual-energy X-ray absorptiometry measurements at 9-10 y of age from the Avon Longitudinal Study of Parents and Children (ALSPAC) Am J Clin Nutr 2007 85 1578 1585 17556696 10.1093/ajcn/85.6.1578

[bib18] Leary SD Davey Smith G Rogers IS Reilly JJ Wells JCK Ness AR Smoking during pregnancy and offspring fat and lean mass in Childhood Obesity (Silver Spring) 2006 14 2284 2293 17189557 10.1038/oby.2006.268 PMC1890311

[bib19] Yang J Loos RJF Powell JE FTO genotype is associated with phenotypic variability of body mass index Nature 2012 490 267 272 22982992 10.1038/nature11401 PMC3564953

[bib20] Frayling TM Timpson NJ Weedon MN A common variant in the FTO gene is associated with body mass index and predisposes to childhood and adult obesity Science 2007 316 889 894 17434869 10.1126/science.1141634 PMC2646098

[bib21] Freathy RM Kazeem GR Morris RW Genetic variation at CHRNA5-CHRNA3-CHRNB4 interacts with smoking status to influence body mass index Int J Epidemiol 2011 40 1617 1628 21593077 10.1093/ije/dyr077 PMC3235017

[bib22] Speliotes EK Willer CJ Berndt SI Association analyses of 249 796 individuals reveal 18 new loci associated with body mass index Nat Genet 2010 42 937 948 20935630 10.1038/ng.686 PMC3014648

[bib23] Craig WY Palomaki GE Haddow JE Cigarette smoking and serum lipid and lipoprotein concentrations: an analysis of published data Br Med J 1989 298 784 788 2496857 10.1136/bmj.298.6676.784 PMC1836079

[bib24] Tony H Chen H Chiu YH Boucher BJ Transgenerational effects of betel-quid chewing on the development of the metabolic syndrome in the Keelung Community-based Integrated Screening Program Am J Clin Nutr 2006 83 688 692 16522918 10.1093/ajcn.83.3.688

[bib25] Laubenthal J Zlobinskaya O Poterlowicz K Cigarette smoke-induced transgenerational alterations in genome stability in cord blood of human F1 offspring FASEB J 2012 10 3946 3956 22730438 10.1096/fj.11-201194

[bib26] Linschooten JO Verhofstad N Gutzkow K Paternal lifestyle as a potential source of germline mutations transmitted to offspring FASEB J 2013 27 2873 2879 23538710 10.1096/fj.13-227694 PMC3688758

[bib27] Barber R Plumb MA Boulton E Roux I Dubrova Y Elevated mutation rates in the germ line of first- and second-generation offspring of irradiated male mice Proc Natl Acad Sci 2002 99 6877 6882 11997464 10.1073/pnas.102015399 PMC124497

