# Supplementary material for: Prepubertal start of father's smoking and increased body fat in his sons: further characterisation of paternal transgenerational responses
Source: Eur J Hum Genet. 2014 Apr 2;22(12):1382–6. doi: 10.1038/ejhg.2014.31 (PMC4085023; doi:10.1038/ejhg.2014.31)
Supplement: Supplementary Tables [file ejhg201431x1.doc]

**Supplementary Table 1: Unadjusted mean BMI in the offspring at various ages restricted to those with data on when father started smoking**

| **Offspring Age** | **Sons** | | | **Daughters** | | |
| --- | --- | --- | --- | --- | --- | --- |
|  | **Mean (sd)** | **n** |  | **Mean (sd)** | **n** |  |
| 7  9  11  13  15  17 | 16.05 (1.85)  17.38 (2.68)  18.70 (3.24)  19.80 (3.24)  20.91 (3.29)  22.46 (3.72) | 3104  2838  2662  2312  1975  1653 |  | 16.32 (2.12)  17.86 (2.93)  19.28 (3.44)  20.65 (3.48)  21.74 (3.58)  22.87 (4.11) | 3012  2864  2686  2353  2173  2087 |  |

**Supplementary Table 2. Adjusted^a^ mean difference (Md) [95% CI] in (a) BMI, (b) waist circumference and (b) fat mass assessed by DXA of the offspring if their father started smoking regularly <11 years of age (those who either did not smoke or did not start until 11 years of age or older are the reference group). *Combining genders***

|  | **Age 7** | **Age 9** | **Age 11** | **Age 13** | **Age 15** | **Age 17** |
| --- | --- | --- | --- | --- | --- | --- |
| **BMI**  N^b^  Md  [95% CI]  p  Waist circ (cm)  N^b^  Md  [95% CI]  P  Fat mass (g)  N^b^  Md  [95% CI]  p | 66  +0.28  [-0.20, +0.77]  0.255  66  +0.36  [+0.07,+0.66]  0.017  Not collected | 52  +0.65  [-0.13,+1.42]  0.101  52  +0.69  [-0.68, +3.52]  0.186  49  +0.92  [-0.50,+2.35]  0.204 | 53  +0.75  [-0.17,+1.67]  0.109  53  +1.37  [-1.17,+3.92]  0.290  52  +0.91  [-0.94, +2.77]  0.334 | 41  +1.99  [+0.93, +3.04]  <0.0001  44  +3.41  [+0.72,+6.32]  0.014  40  +3.87  [+1.38,+6.36]  0.002 | 41  +1.19  [+0.11,+2.28]  0.030  41  +3.63  [+0.81,+6.46]  0.012  36  +2.11  [-0.89,+5.10]  0.168 | 27  +2.39  [+0.88,+3.89]  0.002  Not collected  27  +5.26  [+1.33,+9.19]  0.009 |

^a^Adjusted for parity, parental education, maternal smoking during pregnancy, housing tenure and paternal smoking at conception

^b^Numbers whose fathers started smoking regularly prior to age 11 and had the outcome data.

**Supplementary Table 3: Adjusted^a^ mean (SD) BMI at various ages by age *MOTHER* started smoking.**

| **Age mother started smoking** | **Age 7** | **Age 9** | **Age 11** | **Age 13** | **Age 15** | **Age 17** |
| --- | --- | --- | --- | --- | --- | --- |
| **Sons (n)** | 2891 | 2540 | 2479 | 2158 | 1843 | 1539 |
| <11  11-12  13-14 | 15.89 (0.86)  16.58 (0.37)  16.88 (0.29) | 16.85 (1.27)  17.87 (0.58)  18.75 (0.48) | 17.95 (1.94)  19.65 (0.72)  20.58 (0.59) | 20.24 (1.95)  20.41 (0.80)  21.00 (0.49) | 22.41 (1.57)  21.80 (0.87)  21.89 (0.67) | 24.70 (2.06)  24.27 (1.19)  26.00 (0.98) |
| 15+ | 16.63 (0.25) | 18.12 (0.42) | 19.95 (0.52) | 21.24 (0.38) | 21.56 (0.57) | 28.85 (0.88) |
| Never  P | 16.57 (0.26)  0.350 | 18.02 (0.43)  0.401 | 19.90 (0.53)  0.181 | 21.26 (0.39)  0.520 | 21.53 (0.58)  0.867 | 24.59 (0.89)  0.068 |
| **Daughters (n)** | 2832 | 2690 | 2925 | 2216 | 2044 | 1951 |
| <11 | 16.51 (0.88) | 19.24 (1.48) | 20.82 (1.57) | 21.26 (1.78) | 22.66 (1.52) | 25.14 (2.07) |
| 11 -12 | 16.62 (0.39) | 18.73 (0.59) | 20.37 (0.67) | 22.40 (0.80) | 22.82 (0.84) | 24.89 (0.99) |
| 13-14 | 16.48 (0.26) | 18.35 (0.36) | 19.96 (0.45) | 22.40 (0.80) | 22.42 (0.57) | 24.51 (0.62) |
| 15+ | 16.46 (0.20) | 18.12 (0.29) | 19.75 (0.35) | 20.98 (0.54) | 22.09 (0.47) | 23.74 (0.47) |
| Never  P | 16.44 (0.21)  0.801 | 18.15 (0.30)  0.642 | 19.75 (0.36)  0.604 | 20.92 (0.55)  0.487 | 22.12 (0.47)  0.795 | 24.01 (0.49)  0.286 |

**^a^ Adjusted for parity, parental education, maternal smoking during pregnancy, housing tenure and paternal smoking at conception**

**Supplementary Table 4a: Puberty data: For girls - mean (sd) age at menarche (years) by** age father started smoking regularly.

| **Age father started smoking** | **Mean (SD) age at menarche** |
| --- | --- |
| <11 | 10.66 (4.85) |
| 11-12 | 11.79 (2.83) |
| 13-14 | 10.74 (4.75) |
| 15+  Never | 11.20 (3.93)  11.38 (3.59) |
| F=0.86 (p=0.486) |  |

**Supplementary Table 4b: Proportion of boys at each stage of pubic hair growth at age 11 by age father started smoking.**

| **Age father started smoking** | **Stage 1**  **(39.4%)** | **Stage 2**  **(37.8%)** | **Stage 3, 4 or 5**  **(22.8%)** |
| --- | --- | --- | --- |
| <11 | 10 (45.5%) | 7 (31.8%) | 5 (22.7%) |
| 11-12 | 16 (28.1%) | 26 (45.6%) | 15 (26.3%) |
| 13-14 | 58 (34.7%) | 64 (38.3%) | 45 (26.9%) |
| 15+  Never | 321 (39.7%)  448 (40.3%) | 301 (37.3%)  421 (37.9%) | 186 (23.0%)  242 (21.8%) |
| X^2^=6.55 (p=0.586) |  |  |  |

**Supplementary Table 5: Mean (sd) BMI of father (assessed at time of conception of study child) and age he started smoking regularly**

| **Age father started smoking** | **Fathers of Sons** | | **Fathers of Daughters** | |
| --- | --- | --- | --- | --- |
|  | **Mean BMI (sd)** | **n** | **Mean BMI (sd)** | **n** |
| <11 | 24.25 (3.08) | 62 | 24.74 (3.56) | 53 |
| 11-12 | 24.89 (2.96) | 127 | 25.66 (4.37) | 133 |
| 13-14 | 25.29 (3.72) | 372 | 25.31 (3.65) | 339 |
| 15+  Never | 25.19 (3.22)  25.13 (3.16) | 1528  1879 | 25.23 (3.21)  25.15 (3.34) | 1445  1838 |
|  | F=1.93 (p=0.102) |  | F=2.56 (p=0.217) |  |

**Supplementary Table 6. The proportion of offspring who had ever smoked a whole cigarette by ages 11, 13 and 15 years according to age at paternal onset of smoking (smoking fathers only).**

| **Age father started smoking** | **% (n) smoking at 11 years** | | **% (n) smoking at 13 years** | | **% (n) smoking at 15 years** | |
| --- | --- | --- | --- | --- | --- | --- |
|  | **Boys** | **Girls** | **Boys** | **Girls** | **Boys** | **Girls** |
| **<11 years** | **26.1% (6)** | **15.4% (4)** | **18.2% (4)** | **41.7% (10)** | **45.0% (9)** | **76.9% (20)** |
| **11-12** | **16.2% (11)** | **23.0% (20)** | **19.6% (11)** | **28.9% (22)** | **66.7% (30)** | **56.8% (46)** |
| **13-14** | **13.8% (26)** | **19.7% (38)** | **17.4% (29)** | **26.7% (46)** | **48.5% (66)** | **64.8% (105)** |
| **15+** | **11.6% (111)** | **14.6% (141)** | **19.2% (167)** | **23.3% (203)** | **47.0% (336)** | **59.1% (466)** |
| **P Value** | **0.131** | **0.089** | **0.956** | **0.126** | **0.084** | **0.155** |

**Supplementary Table 7. Adjusted^a^ mean difference (Md) [95% CI] in a) waist circumference and b) fat and lean mass assessed by DXA of the offspring if their father started smoking regularly <11 years of age (those who either did not smoke or did not start until 11 years of age or older are the reference group).**

|  | **Age 13** | **Age 15** | **Age 17** |
| --- | --- | --- | --- |
| **Sons**  Waist circumference  N^b^  Md [95% CI]  P  Fat mass  N^b^  Md [95% CI]  P  Lean mass  N^b^  Md [95% CI]  P | 189  7.75 [3.24, 12.28]  <0.001  19  6.37[2.95,9.80]  <0.0001  19  0.44 [-0.28,1.16]  0.840 | 17  6.08[1.74,10.4]  0.006  17  5.41[1.38,9.45]  0.009  17  0.37[-2.84,3.57]  0.823 | Not collected  8  10.5[3.50,17.3]  0.003  8  -0.37[-4.78, 4.05]  0.871 |
| **Daughters**  Waist circumference  N^b^  Md [95% CI]  P  Fat mass  N^b^  Md [95% CI]  P  Lean mass  N^b^  Md [95% CI]  P | 19  0.57[-3.48,4.63]  0.781  21  1.53[-1.68,4.74]  0.349  21  -0.10[-0.30, 0.49]  0.036 | 19  1.97[-2.10,6.03]  0.340  19  0.89[-4.56,2.77]  0.633  19  1.55[-0.28,3.38]  0.097 | Not collected  13  1.73[-3.50,6.9]  0.514  13  2.35[0.01,4.69]  0.049 |

^a^Adjusted for parity, parental education, maternal smoking during pregnancy, housing tenure and paternal smoking at conception AND child ever smoked a cigarette at that age

^b^Numbers whose fathers started smoking regularly prior to age 11.

**Supplementary Table 8. Adjusted^a^ mean difference (Md) [95% CI] in a) waist circumference and b) fat and lean mass assessed by DXA of the offspring if their father started smoking between 11 and 13 years of age (those who either did not smoke or did not start until >13 years of age or older are the reference group) [Note: those starting < 11 are removed].**

|  | **Age 7** | **Age 9** | **Age 11** | **Age 13** | **Age 15** | **Age 17** |
| --- | --- | --- | --- | --- | --- | --- |
| **Sons**  Waist circumference  N^b^  Md [95% CI]  P  Fat mass  N^b^  Md [95% CI]  P  Lean mass  N^b^  Md [95% CI]  P | 73  0.36[-0.77,1.49]  0.530  Not collected  Not collected | 66  1.25 [0.66,3.10]  0.187  62  0.51[-0.71,1.73]  0.413  62  0.11[-0.87,0.65]  0.774 | 64  1.70[-0.70,4.10]  0.164  61  0.40[-1.20,2.14]  0.580  61  0.66[-1.16,1.04]  0.913 | 64  2.76[0.35,5.18]  0.025  48  0.94[-1.21,3.09]  0.392  48  0.94 [-0.18,1.28]  0.138 | 49  2.25[-0.38,4.88]  0.158  36  -0.26[-3.05,2.54]  0.858  36  1.20[-1.05,3.45]  0.295 | Not collected  31  0.15[-3.38,3.67]  0.936  31  0.05[-2.34,2.24]  0.967 |
| **Daughters**  Waist circumference  N^b^  Md [95% CI]  P  Fat mass  N^b^  Md [95% CI]  P  Lean mass  N^b^  Md [95% CI]  P | 89  0.21[-0.92, 1.32]  0.147  Not collected  Not collected | 86  0.88[-0.88,2.56]  0303  82  0.66[-0.44,1.76]  0.146  82  0.39 [-0.30,1.08]  0.270 | 80  0.62[-1.44,2.67]  0.555  79  0.56[-0.95,2.10]  0.465  79  0.32 [-0.70,1.35]  0.536 | 76  0.55[-1.55,2.65]  0.605  66  0.78[-1.04,2.61]  0.400  66  0.13[-0.27, 0.53]  0.514 | 66  1.51[-0.71,3.74]  0.212  65  0.96[-1.06,2.97]  0.662  65  0.15[-0.85,1.16]  0.763 | Not collected  54  1.09[-1.46,3.65]  0.151  54  0.09[-1.26,1.08]  0.875 |

^a^Adjusted for parity, parental education, maternal smoking during pregnancy, housing tenure and paternal smoking at conception

^b^Numbers whose fathers started smoking regularly between the ages of 11 and 13.

**Supplementary Table 9: Allele risk score: mean (sd) weight allele score by** age father started smoking regularly.

| **Age father started smoking** | **Sons** | | **Daughters** | |
| --- | --- | --- | --- | --- |
|  | **Mean (sd)** | **n** | **Mean (sd)** | **n** |
| <11 | 0.451 (0.253) | 48 | 0.460 (0.036) | 34 |
| 11-12 | 0.452 (0.052) | 105 | 0.451 (0.055) | 104 |
| 13-14 | 0.449 (0.053) | 269 | 0.451 (0.058) | 242 |
| 15 | 0.453 (0.055) | 1187 | 0.451 (0.054) | 1115 |
| Never | 0.449 (0.053) | 1495 | 0.451 (0.054) | 1458 |
|  | F=1.03 (p=0.392) |  | F=0.26 (p=0.905) |  |

**Supplementary Table 10: Unadjusted associations between SNPs rs9939609 (*FTO*) and**

**rs1051730 (*CHRNA5-CHRNA3-CHRNB4*) and age father started smoking regularly.**

| **Sons** | **Never** | **<11** | **11+ years** |
| --- | --- | --- | --- |
| FTO  AA (15.2%)  AT (47.8%)  TT (37.0%)  X^2^ =2.06 (p=0.724) | 249 (15.9%)  749 (47.8%)  569 (36.3%) | 10 (18.2%)  26 (47.7%)  19 (34.5%) | 234 (14.4%)  777 (47.8%)  614 (37.8%) |
| rs1051730  CC (45.6%)  CT (44.0%)  TT (10.4%)  X^2^ =1.78 (p=0.776) | 748 (46.2%)  696 (43.3%)  175 (10.8%) | 25 (42.4%)  27 (45.8%)  7 (11.9%) | 766 (45.2%)  760 (44.9%)  168 (9.9%) |
| **Daughters** |  |  |  |
| FTO  AA (17.0%)  AT (46.6%)  TT (36.3%)  X^2^ =2.66 (p=0.617) | 245 (16.2%)  714 (47.2%)  553 (36.6%) | 9 (24.3%)  15 (40.5%)  13 (35.1%) | 275 (17.7%)  719 (46.2%)  563 (36.2%) |
| rs1051730  CC (45.1%)  CT (43.4%)  TT (11.4%)  X^2^ =3.46 (p=0.484) | 674 (44.5%)  654 (43.2%)  187 (12.3%) | 18 (51.4%)  15 (42.9%)  2 (5.7%) | 709 (45.6%)  686 (43.7%)  166 (10.7%) |
